# National Policies and Older People’s Healthcare in Sub-Saharan Africa: A Scoping Review

**DOI:** 10.5334/aogh.2401

**Published:** 2019-06-26

**Authors:** Sule Saka, Frasia Oosthuizen, Manimbulu Nlooto

**Affiliations:** 1 University of Kwa-Zulu Natal, ZA

## Abstract

**Background:**

Sub-Saharan Africa (SSA) is undergoing a rapid demographic change, with more people reaching old age. There is, however, little information available about healthcare policies with regards to this age group in this region of the world.

**Objectives:**

This scoping review aims to map the healthcare policies in sub-Saharan Africa (SSA) after the 2002 United Nations Madrid International Plan of Action on Ageing (MIPAA) with an eye towards to identifying strategies for promoting older people’s access to health care, integration of older people’s diseases into primary health care and the level of training and research in geriatrics and gerontology in SSA.

**Methods:**

This review adopted Arskey and O’Malley’s five-step methodology for scoping review and used the guide by Levac et al to operationalize the steps. Potentially relevant literature in English published between January 2003 and December 2017 was identified through PubMed, Google Scholar, EBSCOhost, and manual search. Articles that related to ageing in SSA in line with the aims of the review were included. The identified articles were independently assessed by the authors and the decision on the articles to be included was reached by a consensus.

**Findings:**

A total of 363 articles were identified through the databases and manual search of which only 4.7% (17/363) of the articles were included in the review. The findings showed that many SSA countries have formulated policies on healthy ageing and a few have policies to promote access to health care for the older people. The integration of non-communicable diseases (NCDs) management into primary health care (PHC) is encouraging but mental health appears to have been completely neglected. Training and research in gerontology and geriatrics are hardly supported by governments in SSA.

**Conclusions:**

Significant progress has been made by the SSA countries in policy formulation with regards to older persons but not much has been achieved with the implementation of the policies.

## Introduction

An increase in the ageing population is a reality in many sub-Saharan African (SSA) countries. Older people in SSA are defined as those aged 60 years and above [1]. Sub-Saharan Africa, consisting of 48 of the 54 African countries and excluding northern African countries, was home to an estimated 46 million people aged 60 years and above in 2015 [1,2]. This number is projected to increase more than twofold by 2050, indicating that the region will have the fastest growth rate of older people compared to any other region of the world within this period [2,3]. Sub-Saharan Africa is, however, the most impoverished region of the world with most of the countries classified as low-income countries [4].

In realization of the ageing problem, members of the United Nations and the African Union, as well as many governments in SSA, signed the two international frameworks on ageing, the 2002 United Nations Madrid International Plan of Action on Ageing (MIPAA) and the 2003 African Union Policy Framework and Plan of Action on Ageing (AU-Plan). These two international frameworks emphasized three strategies: (1) multidimensional health promotion to prevent disease and disability among the older people; (2) implementation of policies to ensure unrestricted access to adequate rehabilitation and curative care for the older people who already suffer from chronic diseases and disability; and (3) adequate training of the healthcare workforce for the care of older people [5,6]. In order to achieve these goals, the AU including SSA governments proposed two actions for implementation in the African Union Health Strategy 2007–2013 document. The action plans are: (1) To incorporate preventive and curative care services for major NCDs in primary health care; and (2) to improve access to health care for the older people by removing financial barriers and creating provisions for the social protection of older people [7].

However, a decade after the agreements little is known about the progress in the implementation of these policy frameworks in SSA. This review aims to map the policies with regards to the ageing population in SSA since the 2002 MIPAA and the 2003 AU-Plan.

## Methods

This scoping review adopted the five-step methodology for scoping reviews propounded by Arskey and O’Malley [8]. The guide proposed by Levac et al. was used in operationalizing each step [9].

### Step 1: Identification of research questions

This review was intended to examine policies regarding the ageing population in SSA. A preliminary search for policy on healthy ageing in SSA using the terms “older persons”, “healthcare”, and “sub-Saharan Africa” yielded articles on barriers and the vulnerability of older persons. We, therefore, proceeded to identify existing frameworks and policies on healthy ageing. This led to the MIPAA and AU-Plan. We found that these frameworks included socioeconomic, cultural and health agendas; we, however, focused on the health agenda.

Reviewing the MIPAA and AU-Plan, we identified one broad research question as follows: What are the national policies on ageing in SSA post-2002 MIPAA and 2003 AU-Plan agreements? To operationalize the broad research question, the following three research questions, which formed the subtheme of this review, were identified:

1. What are the strategies for the promotion of older people’s access to health care?
2. What is the level of integration of non-communicable diseases and mental health into primary health care in SSA?
3. What is the level of training and research in geriatrics and gerontology in SSA?

### Step 2: Identification of relevant studies

Prior to the literature search, agreement was reached on the inclusion criteria. These criteria were then used to guide the search. The inclusion criteria included:

1. Original research on the subthemes
2. Reviews including narratives, scoping and systematic reviews
3. Governmental and non-governmental reports
4. Published in English
5. January 2003 to December 2017

Exclusion criteria included:

1. Literature without significant findings on any of the sub-themes
2. Articles with abstracts only
3. Thesis
4. Articles from the same authors with similar findings
5. Research articles that duplicate findings in the same country
6. Inaccessible reports published within the study period

### Search Strategies

Publication titles from the preliminary search were reviewed and used to refine the terms for the systematic search. The research questions and key concepts were adopted for the search strategy in the electronic databases. The librarian for the school of pharmacy, University of KwaZulu-Natal identified the relevant keywords and MeSH term and gave advice on relevant databases that could be useful.

The search was conducted using PubMed, EBSCOhost and Sabinet (South African) databases. Keywords and terms such as “Older people in sub-Saharan Africa,” “Ageing policies in sub-Saharan Africa,” “Health care access for older people in Africa,” “Geriatric training in sub-Saharan Africa,” “Research in geriatrics and gerontology in sub-Saharan Africa,” “Non-communicable diseases,” “Primary health care,” and “Mental health in sub-Saharan Africa” were used for the search. A manual search was conducted to identify potential literature that could have been missed during the database search. Relevant references from the identified literature were also searched.

### Step 3: Study selection

The study selection was carried out into two stages. Two reviewers (the primary and secondary author) applied the inclusion and exclusion criteria to the identified reports. Abstracts were first reviewed, and full articles were read after the reviewers agreed on the relevance of the article based on the abstract. The identified full articles were independently assessed by the reviewers and the decision on the articles to be included was reached by a consensus. The researchers developed criteria to streamline articles by prioritization. In the study selection, intergovernmental policy papers were prioritized over individual reports of similar finding, since they provide a pool of information from many studies. The most recent reports of similar findings were included in the review. Articles that compared health care policies of countries in SSA were given preference over articles presenting a similar national policy.

### Step 4: Charting the data

A data-charting form was developed and agreed upon by the reviewers, and this was used to extract data from literature. Information from eligible articles was extracted by the first author and reviewed by the other authors. Information including title, author, year of publication, article type, sub-theme addressed, the country of the study and the key findings were extracted into a data-charting form developed in Microsoft Excel.

### Step 5: Collating, summarizing and reporting results

The descriptive analysis of the articles for each research question was done based on the method of Arkey and O’Malley [8]. The reports were collated based on the thematic analysis. This informed the recommendation for policy implementation and further research.

## Results

**Figure 1:**
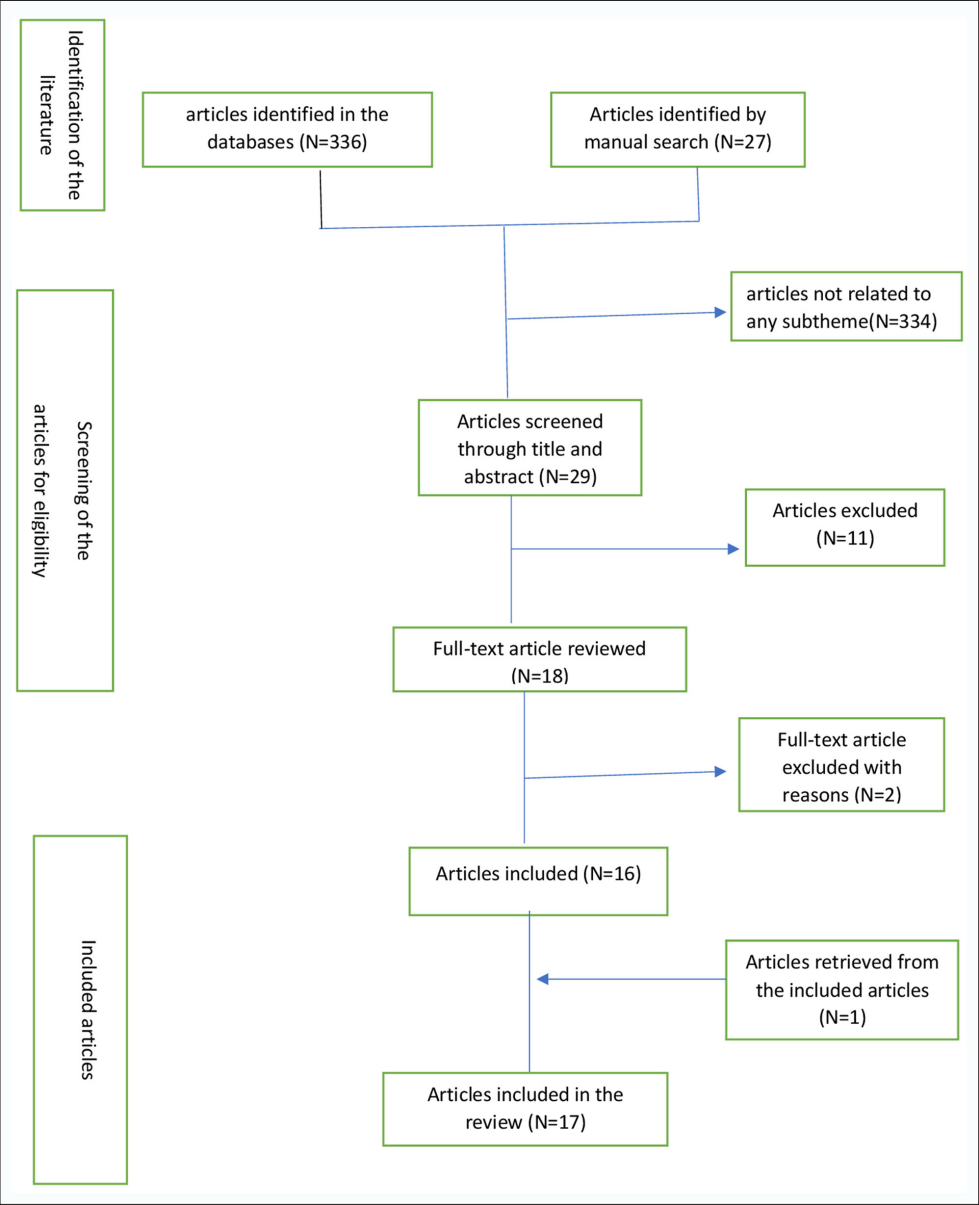
The Flow chart for the selection process.

Figure 1 describes the flowchart of the selection process for the included articles. A total of 336 reports were found after an electronic database search. An additional 27 reports were identified through manual search. Only 5.0% (18/363) articles were considered potentially relevant for the full-text review and 4.7% (17/363) articles that met the eligibility criteria were included in the final review after the synthesis of information in the papers.

Table 1 presents the general characteristics of the included articles. The majority (10/17; 58.8%) of the articles were policy papers, published within a decade of the MIPAA and AU-Plan agreement. The details of the included articles are presented in supplement 1.

**Table 1:** Characteristics of the included articles.

Articles	Number (%) of studies

**Year of publication**	

2003–2008	4 (23.5)
2009–2014	8 (47.1)
2015–2017	5 (29.4)
**Types**	

Policy paper	10 (58.8)
Original research	6 (35.3)
Reviews	1 (5.9)
Sub-theme discussed*	

Health care access for older persons	8
Integration of NCD into primary health care	3
Integration of mental health into PHC	3
Gerontology research and training	8

* *Note*: the total may be more than 17 because a few papers discussed more than one sub-theme.

Table 2 presents the list of countries implementing policy for the ageing population in SSA. More than 8 countries in SSA have legislations and are implementing ageing policies while others such as Nigeria, Cameroon and Rwanda have drafted policies.

**Table 2:** Countries with National legislation on ageing.

Country	Legislation	Year

South Africa	Older Persons Act 2006	2006
Ghana	National Policy on Older People	2009
Mozambique	National Plan for Older People	2005
Uganda	National Plan of Action for Older People	2012
Tanzania	National Ageing Policy	2003
Zimbabwe	Older Person Act	2012
Kenya	National Policy on Older Persons and Ageing (Revised)	2014
Ethiopia	National Plan of Action on Older Persons	2006
Malawi	National Policy for Older Persons	2016

## Discussion

This review showed that countries in SSA, including Ghana, Mozambique, South Africa, Uganda and Tanzania, have ratified national policies on the ageing population, while a few like Nigeria, Cameroon and Rwanda have drafted policies which are awaiting passage into law by their legislatures [10,11,12,13,14]. This observation is consistent with the United Nations Economic Commission for Africa report on the implementation of the MIPAA in Africa [15], and an improvement over a previous report [16]. This, no doubt, is commendable. However, it appears most of the governments lack the political will to implement these policies, considering the long period between the time of legislation and its implementation (as in the case of Zimbabwe Older Persons Act), and the many impediments to the legislation of drafted policies, as exemplified in the revised National Policy on Ageing (2008) in Nigeria [16,17]. Even in the countries that have legislated the policies, meager resources are allocated to the implementation [18].

The current policies to promote access to health care for older people in SSA are not encouraging. With the exception of countries such as Senegal, Ghana and South Africa that have either implemented free healthcare or exempted the older people from paying health insurance premiums, the older people in many other SSA countries are not covered by health insurance [19,20,21,22,23,24,25,26,27,28]. Nigeria, the country with the largest population of older people in SSA, has no health care access policies targeted at older persons and the health insurance is intrinsically designed to exclude this category of the population [29]. The observation of this review is in line with the previous reports [17,30]. The lack or inadequate health insurance coverage for older people will restrict their access to healthcare and thus contributes to preventable deaths among them.

It also appears that only a few countries in SSA, including South Africa, Ghana, Nigeria and Cameroon, have evidence of integration of NCDs treatments into PHC [31,32,33]. This observation, which has earlier been documented [30], indicates a lack of preparedness of the national governments in SSA for an imminent disaster. The World Health Organization estimates in 2015 showed an increasing mortality due to NCDs in SSA and indicated that by 2020, NCDs will be the main cause of morbidity and mortality in the region [17,32]. Already, in many West African countries such as Nigeria and Ghana, NCDs are responsible more for older people’s deaths and admissions to the hospitals than infectious diseases such as tuberculosis and HIV/AIDS [33]. Considering the limited resources available to many governments in SSA, it will be wise to give priority to interventions that aim at preventing and reducing NCDs; this can only be achieved if the NCD management is integrated into PHC.

Mental health appears not to be a health priority in SSA. This review showed that only South Africa and Kenya have incorporated mental health into the national policies on ageing consistent with the WHO reports [16], and even in these two countries, translating the policy into action has received little impetus from the governments and there is evidence that the policy is not achieving the desired goal [34]. Mental health has rarely been integrated into PHC in SSA despite studies that have shown the feasibility and the health benefits of such integration in the region [34,35,36]. According to the 2013 Global Burden of Diseases estimates, mental disorders including depression, schizophrenia and anxiety disorders account for almost 10% of the disease burden in SSA, parallel to 14% global disease burden [37]. The morbidity and mortality from NCDs and mental disorders no doubt will take a great toll on the meager health care resources in many SSA countries. The healthcare systems in SSA therefore need to be responsive and adopt a course approach that is built on PHC.

Countries in SSA including Cameroon, Kenya, South Africa and Nigeria have plans in their national ageing policies to promote training in gerontology for health care providers and the development of a cadre of academic geriatricians [10,11,13,17,31]. The policies also highlighted the establishment and funding of geriatric research institutions, although this has largely not been translated into action, and government support for institutions offering geriatric training is almost non-existent except in countries such as Kenya and South Africa [38]. The number of institutions offering courses in geriatrics and gerontology in SSA is increasing although it is still grossly inadequate. This review showed an improvement in the number of institutions offering geriatric training compared to a previous report [39].

Despite the challenges, researchers in SSA continue to make significant contributions, judging from the number of published articles on geriatrics and gerontology in peer-reviewed journals [40,41]. However, this achievement is still not adequate to fill the information gap necessary for effective policy formulation and action on healthy ageing in the SSA. Most of the research on ageing in SSA has focused on healthcare accessibility [23,24,25,26], while studies that can convincingly influence government policy are lacking. This review observed that there is a dearth of studies on the reasons for the slow implementation of the ageing policies and the economic and human loss associated with the non-implementation of the policies. Economic data is a major factor that can influence the decision of policy makers especially when setting priorities and making decisions in the face of competing national interests [42].

## Limitations

This review did not consider publications in French, which is a major language spoken in some countries in SSA particularly in West Africa. Consequently, national policies and local perceptions of issues affecting healthy ageing in these nations might have been omitted. The review also did not cover articles published in books; thus, the possibility exists that some critical contributions to the discussions could have been missed.

## Conclusions

Although progress has been made by SSA in policy formulation on healthy ageing, not much has been achieved with the implementation of these policies. Access to healthcare for older persons in the region remains limited. Many countries in SSA appear to be striving towards the integration of NCDs into PHC but there is generally a low priority for mental health while support for training and research on geriatrics and gerontology is poor. Sub-Saharan Africa needs to prioritize health care of older people in their developmental plans. Training of healthcare personnel on the care of older people should also be given an impetus in the region. A regional collaboration in this regard is advocated.

## Additional File

The additional file for this article can be found as follows:

10.5334/aogh.2401.s1 Supplement 1. The included articles.

## References

[1] United Nations. World population ageing report 2015 Department of economic and social affairs, population division. New York.

[2] The World Bank. Sub-Saharan Africa/Data. https://data.worldbank.org/region/sub-saharan-africa. Accessed September 23, 2018.

[3] United Nation Population Division. World population prospects: The 2012 revision. http://esa.un.org/unpd/wpp/index.htm. Accessed December 20, 2013.

[4] Aboderin IA and Beard J. Older people’s health in sub-Saharan Africa. 2015; 385: e9–e11. DOI: 10.1016/S0140-6736(14)61602-0 25468150

[5] United Nations. Madrid international plan of action on ageing (MIPAA) New York: United Nations; 2002.

[6] African Union/Help Age International. Policy framework and plan of action on ageing Nairobi: HAI African Regional Development Centre; 2003.

[7] African Union. African health strategy 2007–2013. Third session of the African Union conference of ministers of health Johannesburg, South Africa; 2007.

[8] Arksey H and O’Malley L. Scoping studies: Towards a methodological framework. Social Research Methods. 2005; 8: 19–31. DOI: 10.1080/1364557032000119616

[9] Levac D, Colquhoun H and O’Brien KK. Scoping studies: Advancing the methodology. Implementation Science. 2010; 5: 69 DOI: 10.1186/1748-5908-5-69 20854677 PMC2954944

[10] South African Older People’s Act 2006. Ministry of Justice. Available http://www.justice.gov.za/legislation/acts/2006-013_olderpersons.pdf. Accessed July 18, 2017.

[11] Ministry of Labour Social Security and Services. National policy on older people and ageing (revised), 2014 Republic of Kenya Available: http://www.partners-popdev.org/ageing/docs/National_Policy_on_Older_Persons_and_Ageing_Kenya.pdf. Accessed 22 July, 2017.

[12] The Republic of Uganda. National plan of action for older people 2012/2013-2016/2017. Ageing with security and dignity. 2012 Available http://www.mglsd.go.ug/Plans/NATIONAL%20PLAN%20OF%20ACTION%20FOR%20OLDER%20PERSONS.pdf. Accessed 25 August, 2017.

[13] Ministry of Employment and Social Welfare. National ageing policy. Ageing with security and dignity Ghana; 2010 Available https://s3.amazonaws.com/ndpc-static/pubication/Ageing+Policy_July+2010.pdf. Accessed June 17, 2017.

[14] Ministry of Labour Youth Development and Sports. National ageing policy 2003 United Republic of Tanzania Available http://interactions.eldis.org/sites/interactions.eldis.org/files/database_sp/Tanzania/National%20Ageing%20Policy/NAP.pdf. Accessed July 18, 2017.

[15] United Nations Economic Commission for Africa. The third review and appraisal cycle of the implementation of the Madrid international plan of action on ageing in Africa for the period 2012-2017. Final report. Available http://www.un.org/development/desa/ageing/wp…/sites/…/2017/…/eca-report-mipaa2017.pdf. Accessed September 25, 2018.

[16] United Nations Population Fund and HelpAge International. Overview of available policies and legislation, data and research, and institutional arrangements relating to older people—Progress since Madrid Report compiled in preparation for the state of the world’s older people 2012. New York; 2011.

[17] World Health Organization. Healthy ageing in the African region: Situation analysis and way forward. AFR/RC63/4 Documents for the sixty-third session of the WHO regional committee for africa (2013). Available http://www.afro.who.int/index.php?option=com_docman&task=doc_download&gid=8575&Itemid=2593. Accessed October 15, 2016.

[18] Aboderin I. Understanding and advancing the health of older populations in sub-Saharan Africa: Policy perspectives and evidence needs. Public Health Reviews. 2010; 32(2): 357–376. DOI: 10.1007/BF03391607

[19] Scholten F, Mugisha J, Seeley J, et al. Health and functional status among older people with HIV/AIDS in Uganda. BMC Public Health. 2011; 11: 886 DOI: 10.1186/1471-2458-11-886 22111659 PMC3256234

[20] Ssengooba F, Rahman S, Hongoro C, et al. Health sector reforms and human resources for health in Uganda and Bangladesh: Mechanism of effect. Human Resour Health. 2007; 5: 3 DOI: 10.1186/1478-4491-5-3 PMC1800303 17270042

[21] Nabalamba A and Chikoko M. Aging population challenges in Africa. African Development Bank, Chief Economist Complex. 2011.

[22] Exavery A, Klipstein-Grobusch DC, Debpuur C (eds.). Self-rated health and healthcare utilization among rural older people Ghanaians in Kassena-Nankana district 2010: Working paper. Presented in session 59: Trends, patterns, and consequences of non-communicable diseases in Africa. Sixth Union for African Population Studies Conference (UAPS) 2011 Ghana: Navrongo Health Research Centre (NHRC) Accessed August 25, 2013. Available from: http://uaps2011.princeton.edu/papers/110332.

[23] Lutala MP, Kwalya TM, Kasagila EK, Watongoka LH and Mupenda BW. Health care seeking and financial behaviours of the older people during wartime in Goma, Democratic Republic of Congo. African journal of primary health care & family medicine. 2010; 2(1). DOI: 10.4102/phcfm.v2i1.108

[24] Molete M, Yengopal V and Moorman J. Oral health needs and barriers to accessing care among the older people in Johannesburg. South African Dental Journal. 2014; 69(8): 352–7. 26548224

[25] Wandera SO, Kwagala B and Ntozi J. Determinants of access to healthcare by older persons in Uganda: A cross-sectional study. International Journal for Equity in Health. 2015; 14: 26 DOI: 10.1186/s12939-015-0157-z 25889558 PMC4354736

[26] Mulumba M, Nantaba J, Brolan C, Ruano A, Brooker K and Hammonds R. Perceptions and experiences of access to public healthcare by people with disabilities and older people in Uganda. International Journal for Equity in Health. 2014; 13: 76 DOI: 10.1186/s12939-014-0076-4 25928896 PMC4188877

[27] Ka O, Leye M, Gaye A, Sow P, Diop S and Sow A. Towards a geriatrics policy integrated to the primary health cares in Africa (the case of Senegal). Journal of Gerontology & Geriatric Research. 2016.

[28] Parmar D, Williams G, Dkhimi F, Ndiaye A, Asante FA, Arhinful DK, et al. Enrolment of older people in social health protection programs in West Africa—Does social exclusion play a part? Social Science & Medicine. 2014; 119: 36–44. DOI: 10.1016/j.socscimed.2014.08.011 25137646

[29] Mohammed S, Bermejo JL, Souares A, Sauerborn R and Dong H. Assessing responsiveness of health care services within a health insurance scheme in Nigeria: Users’ perspectives. BMC health services research. 2013; 13(1): 1 DOI: 10.1186/1472-6963-13-502 24289045 PMC4220628

[30] Aboderin I. African research on ageing network (AFRAN) policy-research dialogue: Advancing health service provision for older persons and age-related noncommunicable disease in sub-Saharan Africa: Identifying key information and training needs. Reports and outcomes. 2008.

[31] National Population Commission 2015. Nigeria’s 2004 national policy on population for sustainable development. Available https://www.healthpolicyproject.com/pubs/821_FINALNPPReport.pdf. Accessed July 21, 2017.

[32] World Health Organization. African region. Regional framework for integrating essential noncommunicable disease services in primary health care. AFR/RC67/12. Available https://afro.who.int/sites/default/files/2017-08/AFR-RC67-12%20Regional%20framework%20to%20integrate%20NCDs%20in%20PHC.pdf. Accessed September 23, 2018.

[33] Aikins Ad-G, Boynton P and Atanga LL. Developing effective chronic disease interventions in Africa: Insights from Ghana and Cameroon. Globalization and Health. 2010; 6(1): 1 DOI: 10.1186/1744-8603-6-6 20403170 PMC2873935

[34] Jenkin R, Kiima D, Okonji M, Njenga F, Kingora J and Lock S. Integration of mental health into primary care and community health working in Kenya: Context, rationale, coverage and sustainability. Mental Health & Family Medicine. 2010; 7(1): 37–47. PMC2925163 22477921

[35] Davies T and Lund C. Integrating mental health care into primary care systems in low- and middle-income countries: Lessons from PRIME and AFFIRM. Global Mental Health (Camb). 2017; 4: e7 DOI: 10.1017/gmh.2017.3 PMC5454718 28596908

[36] Gureje O, Abdulmalik J, Kola L, Musa E, Yasmy MT and Adebayi K. Integrating mental health into primary care in Nigeria: Report of a demonstration project using the mental health gap action programme intervention guide. BMC Health Services Research. 2015; 15: 242 DOI: 10.1186/s12913-015-0911-3 26094025 PMC4475323

[37] GBD 2013. Mortality and causes of death collaborators global, regional, and national age-sex specific all-cause and cause-specific mortality for 240 causes of death, 1990–2013: A systematic analysis for the global burden of disease study. Lancet. 2014 Published online Dec 17, 2013. DOI: 10.1016/S0140-6736(14)61682-2 PMC4340604 25530442

[38] National Research Council. Ageing in sub-Sub-Saharan Africa: Recommendation for furthering research. Panel on policy research and data needs to meet the challenge of ageing in Africa Committees on Population Division of Behaviourial and Social Sciences and Education 2006. Washington, DC: The National Academies Press.

[39] Dotchin CL, Akinyemi RO, Gray WK and Walker RW. Geriatric medicine: Services and training in Africa. Age and ageing. 2013; 42(1): 124–8. DOI: 10.1093/ageing/afs119 23027519

[40] Frost L, Navarro AL, Lynch M, et al. Care of the elderly: Survey of teaching in an aging Sub-Saharan Africa. Journal Gerontology & Geriatrics Education. 2015; 36(1): 39 DOI: 10.1080/02701960.2014.925886 24884474

[41] United Nations. Directory of research on ageing in Africa 2004–2015. Available http:www.un.org/en/…/publications/pdf/…/Dir_Research_Ageing_Africa_202004-2015. Accessed July 27, 2017.

[42] Tomlinson M and Lund C. Why does mental health not get the attention it deserves? PLoS Medicine. 2012; 9: e1001178 DOI: 10.1371/journal.pmed.1001178 22389632 PMC3289587

