# Supplementary material for: National Policies and Older People’s Healthcare in Sub-Saharan Africa: A Scoping Review
Source: Ann Glob Health. 2019 Jun 26;85(1):91. doi: 10.5334/aogh.2401 (PMC6634477; doi:10.5334/aogh.2401)
Supplement: Supplement 1. — The included articles. [file agh-85-1-2401-s1.pdf]

Supplement 1: The included articles

Author (year)*	Country	Sub-theme evaluated	Comments
World Health Organization (2013)*[17]	Multinational	National ageing policy	Identified little progress in geriatrics and gerontology and lack of political will and commitment on the part of SSA countries.
Aboderin (2008)*[30]	Multinational	Ageing policy and integration of NCDs into PHC	The healthcare system in SSA is still largely inaccessible to older population, although efforts have been made through various methods to provide free or subsidized care. NCD not fully incorporated into PHC, though it is recognized as the solution.
National Research Council (2006)*[38]	Multinational	Ageing research	There is a dearth of research on older people's health and little financial support for the higher education offering geriatrics from the national governments in SSA.
United Nations, Population Fund Help-Age International (2011)*[16]	Multinational	National policies and ageing research, mental health care	A few countries have adopted ageing policy but there is minimal implementation across the spectrum in SSA. The policy is not backed up with budgetary provisions in almost all the countries in SSA.
Frost et al. (2015)*[40]	Multinational	Geriatric training	A large number of the higher institutions in SSA are not offering geriatrics. Funding, lack of expertise and lack of priority of geriatrics in National policy are some of the barriers highlighted.
Dotchin et al. (2013)*[39]	Multinational	Geriatric training	There are few geriatricians in SSA. More investment is needed in the training of healthcare workers for older people's care.
Ka et al. (2016)*[27]	Senegal	Geriatric training	Geriatric courses are being included in the training of healthcare workers in Senegal.
Parmar et al. (2014)*[28]	Ghana and Senegal	Health care access	There are still barriers to healthcare access, especially among the older people in rural areas, ethnic minority and women in Ghana and Senegal, despite NHIS and Sesame Plan.
United Nations (2015)*[41]	Multinational	ageing research	Ageing research in SSA has grown steadily from 13 in 2004 to 85 in 2013, with South Africa and Nigeria producing 29% and 13% of the output respectively. However, research into mental health and healthcare workers' training in geriatrics were almost non-existent.
Jenkin et al. (2010)*[34]	Kenya	Integration of mental health into PHC	Efforts to promote integration of mental health into PHC in Kenya based on evidence and social context.
Gureje et al. (2015)*[36]	Nigeria	Integration of mental health into PHC	There is a lack of coordination of mental health services and policy in Nigeria.
Government of Ghana (2010)*[13]	Ghana	Ageing policy (health care access)	Provision of adequate access to healthcare for older persons, integration of NCDs into PHC and training of healthcare workers.
Ministry of Labour, Social Security and Services (2014)*[11]	Kenya	Ageing policy (Health care access, mental health	Policy to improve mental health, access to care and geriatric training.
Republic of South Africa (2006)*[10]	South Africa	Ageing policy (Health care access, mental health and integration of NCD into PHC)	A comprehensive policy to address access to healthcare, support for geriatric training, and mental health care provisions.
The Republic of Uganda (2012)*[12]	Uganda	Ageing policy (Health care access)	Policy to improve access to health care for the older persons.
Ministry of Labour (2003)*[14]	Tanzania	Health care access and geriatrics training	Policy to improve access to health care for the older people, and research into geriatrics and gerontology.
National Population Commission of Nigeria (2015)*[31]	Nigeria	Integration of NCD into PHC	Provides frameworks for the integration of NCD into PHC

*Citation number in the reference.
